# Cardiac Assessment and Takotsubo-stunning among COPD-exacerbations in-Hospital (CATCH study): when the lungs break your heart—protocol for a prospective observational cohort study

**DOI:** 10.1136/bmjresp-2025-004079

**Published:** 2026-04-29

**Authors:** Rickard Zeijlon, Sara Zooq, Angela Poller, Lowie Vanfleteren, Linnea Molander, Axel Andersson, Mazdak Tavoly, Peter Hällgren Nordhage, Emma Westerlind, N David Åberg, Johan Lönnbro, Micael Oliveira Diniz, Kanya Said, Per Thysell, Sandeep Jha, Tore Hedbäck, Roxana Mincheva, Johan-Emil Bager

**Affiliations:** 1 Department of Acute Medicine and Geriatrics, Sahlgrenska University Hospital, Gothenburg, Region Västra Götaland, Sweden; 2 Department of Molecular and Clinical Medicine, Institute of Medicine, Sahlgrenska Academy, University of Gothenburg, Gothenburg, Sweden; 3 Department of Clinical Physiology, Sahlgrenska University Hospital, Gothenburg, Region Västra Götaland, Sweden; 4 Department of Internal Medicine and Clinical Nutrition, Institute of Medicine, Sahlgrenska Academy, University of Gothenburg, Gothenburg, Sweden; 5 Department of Respiratory Medicine, University Hospital Ghent, Ghent, Belgium; 6 Department of Cardiology, Sahlgrenska University Hospital, Gothenburg, Region Västra Götaland, Sweden; 7 Department of Research, Østfold Hospital Kalnes, Grålum, Norway; 8 Department of Pharmacology, Institute of Neuroscience and Physiology, Sahlgrenska Academy, University of Gothenburg, Gothenburg, Sweden; 9 Department of Radiology, Institute of Clinical Sciences, Sahlgrenska Academy, University of Gothenburg, Gothenburg, Sweden; 10 Department of Radiology, Sahlgrenska University Hospital, Gothenburg, Region Västra Götaland, Sweden; 11 Department of Clinical Sciences, Lund University, Lund, Sweden

**Keywords:** COPD Exacerbations, COPD Pathology, Imaging/CT MRI etc, Patient Outcome Assessment, Pulmonary Disease, Chronic Obstructive, COPD

## Abstract

**Introduction:**

Acute exacerbation of chronic obstructive pulmonary disease (AECOPD) may cause stress-induced transient acute cardiac dysfunction through myocardial stunning, in the form of exacerbation-triggered Takotsubo syndrome (referred to as Takotsubo stunning). Although prior studies suggest an association between AECOPD and transient cardiac dysfunction, existing evidence is limited to retrospective cohorts, case reports and expert consensus. Therefore, the incidence and clinical impact of Takotsubo stunning with acute heart failure (AHF) during AECOPD remain unknown and may be overlooked due to overlapping clinical symptoms. Cardiac Assessment and Takotsubo-stunning among COPD-exacerbations in-Hospital (CATCH study) aims to determine the incidence of Takotsubo stunning during AECOPD and to evaluate its clinical implication.

**Methods and analysis:**

CATCH is a prospective observational cohort study enrolling adults (≥18 years) admitted for AECOPD at Sahlgrenska University Hospital (Gothenburg, Sweden). Participants with chronic left ventricular systolic dysfunction (left ventricular ejection fraction <50%), pre-existing chronic regional wall motion abnormalities (RWMA) or prior type 1 myocardial infarction are excluded. Following informed consent, participants undergo echocardiographic screening for RWMA and/or systolic left ventricular dysfunction. Screening-positive patients have follow-up echocardiography at 24 hours (±6) and 30 days (±48 hours). Those with reversible dysfunction constitute the CATCH case group, while screening-negative participants serve as controls. Additional assessments include ECG, chest X-ray, N-terminal pro-B-type natriuretic peptide blood analysis and COPD severity. Primary outcomes include the incidence of reversible RWMA or left ventricular dysfunction (proxy for Takotsubo stunning) and in-hospital clinical signs of AHF (Killip class >1). A sample size of 150 patients is required for detecting AHF differences (α=0.05, 80% power).

**Ethics and dissemination:**

The study received ethical approval from the Swedish Ethical Review Authority. All participants provided written informed consent. Results will be disseminated through peer-reviewed journals and scientific meetings.

**Registration details:**

The CATCH study is registered at ClinicalTrials.gov (NCT06597331). The reference number for ethical approval is 2024-02071-01 (with addenda 2024-05448-02 and 2025-05861-02).

**WHAT IS ALREADY KNOWN ON THIS TOPIC**

- Severe acute exacerbation of chronic obstructive pulmonary disease (AECOPD) is associated with increased short-term risk of acute cardiovascular events. Retrospective cohorts, case series and expert consensus have indicated an association between AECOPD and stress-induced myocardial stunning, manifesting as exacerbation-triggered Takotsubo syndrome (referred to as Takotsubo stunning in the protocol), which is characterised by acute heart failure that may further aggravate the AECOPD episode.

**WHAT THIS STUDY ADDS**

- Cardiac Assessment and Takotsubo-stunning among COPD-exacerbations in-Hospital (CATCH study) is the first study to systematically test the association between AECOPD and Takotsubo stunning in a prospective cohort.

**HOW THIS STUDY MIGHT AFFECT RESEARCH, PRACTICE OR POLICY**

- The CATCH study will determine the true association between AECOPD and Takotsubo stunning, which will impact AECOPD management.

## Introduction

 This paper describes the protocol of the Cardiac Assessment and Takotsubo-stunning among COPD-exacerbations in-Hospital (CATCH study), investigating to what extent acute exacerbation of chronic obstructive pulmonary disease (AECOPD) triggers cardiac dysfunction through stress-induced myocardial stunning.

AECOPD may cause acute heart failure (AHF) due to stress-induced myocardial stunning, which is probably underdiagnosed (or possibly undiagnosed) in clinical routine.^1 2^ Myocardial stunning was originally defined as temporary cardiac mechanical dysfunction that persists after resolution of ischaemia, with the absence of irreversible histological damage,^3^ and has later been shown to also occur outside of the ischaemic setting.^4^ Elements of myocardial stunning are associated with several conditions, such as after acute myocardial infarction, postcardiac arrest and in the setting of acute myocarditis. However, the Takotsubo syndrome (TS) likely represents a relatively pure form of myocardial stunning, making this condition particularly attractive for investigating stunning per se.^5^

TS is a heart failure syndrome associated with severe emotional or physical stress predominately affecting females (>90% females) in the postmenopausal ages. TS is characterised by rapid onset of reversible left ventricular systolic dysfunction, typically affecting the apical parts of the left ventricle (with full recovery within days to weeks in most cases). Although the pathophysiology of TS is incompletely understood, catecholamine excess (‘catecholaminergic storm’) is thought to play a large role, leading to cardiac microvascular dysfunction and/or direct catecholamine toxicity on the cardiomyocyte, resulting in myocardial stunning.^3 5 6^

Because of its association with stress, TS is sometimes termed ‘stress cardiomyopathy’. However, ‘stress cardiomyopathy’ was first described in 1980,^7^ before the first description of TS in 1990.^8 9^ Furthermore, left ventricular dysfunction in the setting of neurological disease or cerebrovascular insult was described as early as 1908 and later termed neurogenic stunned myocardium, a condition similar to TS, leading some authors to conclude that these entities likely constitute the same syndrome.^10^ The link between these conditions is the association between emotional or physical stressors, leading to acute cardiac dysfunction, probably through myocardial stunning.

Acute pulmonary conditions constitute over one-fifth of all physical triggers of TS and are associated with a more severe in-hospital course and worse long-term outcome, compared with patients with TS without acute pulmonary conditions.^1 11^ There are reports describing patients experiencing frequently recurrent TS triggered by AECOPD,^12 13^ and COPD is significantly over-represented among patients with TS compared with the general population.^14^ Furthermore, patients with COPD hospitalised for TS have a higher incidence of cardiogenic shock, longer length of stay and higher in-hospital mortality compared with those without COPD.^15^ Concurrent respiratory disease such as AECOPD complicates the diagnosis of TS, since the cardiac condition may be masked by the pulmonary symptoms.^14^ Also, the respiratory symptoms may be further exacerbated by (perhaps undiagnosed) myocardial stunning resulting in heart failure.^1 11 14 15^

The term ‘Takotsubo-stunning’ has been suggested for the myocardial stunning that occurs in TS.^16^ This term will also be used in this protocol, referring to the myocardial stunning that occurs in TS. The association between AECOPD and Takotsubo stunning has not been systematically evaluated in a prospective cohort. The purpose of the CATCH study is to improve our understanding of the impact of Takotsubo stunning in patients with AECOPD, thereby potentially enhancing the quality of care.

## Methods and analysis

### Research aim

To investigate the extent to which AECOPD triggers Takotsubo stunning in admitted patients, and to evaluate its subsequent effect on clinical outcomes.

### Objectives and outcomes

#### Primary objectives

1. To determine the incidence of reversible left ventricular systolic dysfunction (as a proxy for Takotsubo stunning) in patients hospitalised for AECOPD at Sahlgrenska University Hospital (Gothenburg, Sweden).
2. To investigate in-hospital clinical manifestations of AHF (defined as Killip class >1, table 1) in patients with signs of Takotsubo stunning versus those without, among patients hospitalised for AECOPD at Sahlgrenska University Hospital.

**Table 1:** Killip classification of acute left-sided heart failure, based on clinical signs supported by chest imaging

Killip class I	No clinical/radiological signs of heart failure.
Killip class II	Clinical and radiological signs of heart failure but not pulmonary oedema (distinct bilateral rales or crackles over lungs suggesting interstitial fluid supported by interstitial fluid on chest X-ray*).
Killip class III	Clinical picture of frank pulmonary oedema supported by radiological* signs.
Killip class IV	Cardiogenic shock or hypotension (measured as systolic blood pressure lower than 90 mm Hg) and evidence of peripheral vasoconstriction (oliguria, cyanosis or sweating).

* Pulmonary ultrasound may be used to confirm Killip class.

#### Secondary objectives

The secondary objectives include comparisons between patients with AECOPD with and without evidence of Takotsubo stunning. These analyses will be conducted in a hypothesis-generating framework to present new possible mechanistic pathways and clinical associations, focusing on the following variables:

- In-hospital and 1-year all-cause mortality, in-hospital complications (acute myocardial infarction, stroke, pulmonary embolism (PE), respiratory failure, clinical deterioration leading to intensive care unit admission) and length of hospital stay.
- ECG (ST elevation, ST depression, QT prolongation, T-wave inversion).
- N-terminal pro-B-type natriuretic peptide (NT-proBNP) in plasma.
- Presenting symptoms (prevalence of chest pain, severity of dyspnoea).
- Echocardiographic signs of right ventricular strain.

#### Tertiary objectives

The tertiary objectives include investigations within three distinct groups of AECOPD: (1) patients with evidence of Takotsubo stunning, (2) patients without evidence of Takotsubo stunning and (3) the overall cohort, including both patients with and without Takotsubo stunning. In accordance with the secondary objectives, these analyses will also be conducted within a hypothesis-generating framework.

- Concordance between echocardiography, chest X-ray and NT-proBNP in displaying signs of heart failure.
- Prevalence of right ventricular pathology.
- Prevalence of diastolic dysfunction.
- The association between NT-proBNP and in-hospital outcome.
- The association between Killip classification and in-hospital outcome.

### Study design overview

CATCH is a prospective observational cohort study of patients admitted to Sahlgrenska University Hospital for AECOPD. COPD is defined as a documented physician diagnosis of COPD in the hospital or primary care medical record. AECOPD is defined according to the 2024 Global Initiative for Obstructive Lung Disease (GOLD) report as: increased dyspnoea and/or cough and sputum that worsens in <14 days which may be accompanied by tachypnoea and/or tachycardia and is often associated with increased local and systemic inflammation caused by infection, pollution or other insult to the airways.^17 18^ Patients meeting all inclusion criteria and no exclusion criteria are eligible for inclusion and screening for echocardiographic signs of regional wall motion abnormality (RWMA) and/or left ventricular systolic dysfunction. Inclusion takes place from Monday to Friday, and each patient can be included only once; repeat admissions for AECOPD will not lead to reinclusion.

### Study population and eligibility criteria

#### Inclusion criteria

1. Adult patients (≥18 years old) admitted to a hospital ward for AECOPD.
2. Inclusion within 72 hours of admission.
3. Written informed consent (IC).

#### Exclusion criteria

1. Prior documented type 1 acute myocardial infarction or documented pre-existing chronic RWMA or reduced left ventricular ejection fraction (LVEF <50%).
2. Expected inability to comply with the protocol (eg, dementia).
3. Previously included in the CATCH study during a prior event with admission for AECOPD.

### Cardiac screening

**Figure 1:**
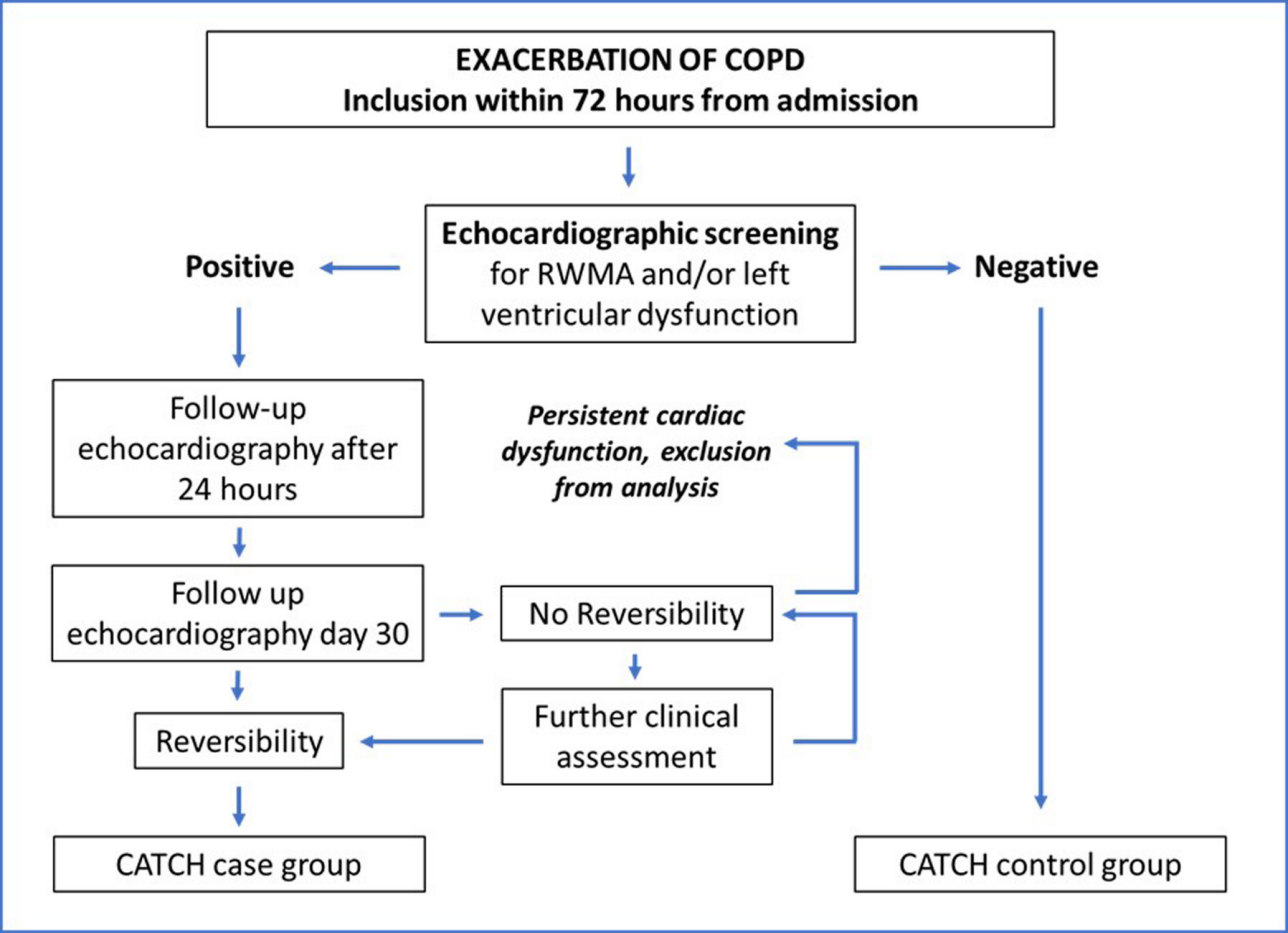
Screening algorithm. CATCH, Cardiac Assessment and Takotsubo-stunning among COPD-exacerbations in-Hospital; COPD, chronic obstructive pulmonary disease; RWMA, regional wall motion abnormality.

After IC, patients are screened with echocardiography (following the algorithm in figure 1) for signs of RWMA and/or left ventricular systolic dysfunction (standard clinical protocol) at the Department of Clinical Physiology at Sahlgrenska University Hospital. The echocardiographic examinations are performed by experienced cardiac sonographers and analysed by experienced clinical physiologists. Negative and positive screening will be defined as follows:

Positive=presence of any RWMA and/or left ventricular systolic dysfunction (LVEF <50%).

Negative=absence of RWMA and no left ventricular systolic dysfunction (LVEF ≥50%).

For patients with a positive screening result, echocardiography will be repeated after 24 (±6) hours and at 30 days (±48 hours). Within the screening-positive group, patients who demonstrate reversibility on repeat echocardiography will constitute the CATCH case group (Takotsubo stunning group), provided that no alternative cause of transient cardiac dysfunction has been identified (according to diagnostic criteria for Takotsubo^16 19^). Reversibility will be defined as normalisation or significant improvement in left ventricular systolic function, indicated by resolution of left ventricular akinesia/hypokinesia and/or improved LVEF ≥10 percentage points.

Cardiac dysfunction detected through study screening will be investigated based on clinical findings and symptoms as per clinical routine, to address alternative causes to Takotsubo stunning. For patients with ongoing or easily provoked chest pain, timely coronary angiography is mandatory to exclude coronary artery occlusion. To exclude or confirm other causes, such as myocarditis or chronic coronary syndromes, cardiac MRI or myocardial perfusion scintigraphy, respectively, may be used according to clinical routine. Hence, in cases with reversible left ventricular dysfunction, Takotsubo stunning will be validated based on clinical investigation together with diagnostic criteria for TS.^19^

If patients within the screening-positive group do not display reversibility by day 30±48 hours (or through further assessment during clinical routine follow-up within 6 months, figure 1), the RWMA and/or left ventricular systolic dysfunction will be considered persistent (ie, fulfilling exclusion criteria unknown at time of inclusion) and the patient will be excluded from analysis.

Patients who turn out negative in screening will constitute the CATCH control group.

### Study procedures

#### Baseline questionnaire

All included patients are interviewed according to a study-specific questionnaire regarding presenting symptoms as well as occurrence and intensity of chest pain and/or breathing difficulties.

#### Echocardiography

The echocardiographic screening protocol has been presented above (study design overview, cardiac screening). All echocardiographic views obtained are saved in the patient’s medical chart, and echocardiographic data according to standard clinical protocol are registered for study purposes. For patients with positive screening results, or where secondary incidental findings emerge, the need for further cardiac investigation/intervention will be assessed in collaboration with the cardiology department.

#### ECG and chest X-ray

Examinations with ECG and chest X-ray are performed in hospital the same day/days as echocardiography. These procedures are performed in accordance with clinical routine.

#### Blood samples

Outside of clinical routine, blood sampling for analysis of NT-proBNP is performed in hospital on the same day/days as echocardiography. If separate specific IC is signed, blood sampling for biobanking will also be obtained at day 0 for metabolomics, proteomics, microRNA and messenger RNA analyses. When available, baseline laboratory work-up from clinical routine is collected regarding troponin I, NT-proBNP, creatinine, haemoglobin, white cell count, C-reactive protein (CRP), electrolytes (sodium, potassium, calcium) and venous/arterial blood gases.

#### Killip classification

Killip class will be assessed using the contemporary Killip classification (table 1), incorporating clinical findings together with chest imaging.^20 21^ Pulmonary ultrasound will be used to assess the presence of interstitial fluid in a subset of patients where Killip class is uncertain and needs further confirmation.

### Collection of data

After IC, data as declared below are collected (also summarised in table 2, schedule of events). If investigations have been performed after contact with healthcare but before IC (ie, after arrival of ambulance/arrival to emergency department/hospital ward but before IC), these data may be included as study data provided that the IC is signed, to avoid unnecessary repeats of investigations/procedures.

**Table 2:** Schedule of events

Schedule of events	Admission	Inclusion (day 0) (within 72 hours of admission)	Day 1 24±6 hours*	Day 2 48±12 hours*	Day 3 72±12 hours*	In hospital (including date)	Postdischarge within 6 months
Eligibility criteria and informed consent		X					
Time from admission to inclusion		X					
GOLD grade (1–4) and group (A, B, E)		X					X
Severity of COPD exacerbation (mild, moderate, severe)^18^		X					
Baseline characteristics including baseline medication	X	X					
Blood pressure and heart rate	X	X	X	X	X		X
Pulse oximetry and respiratory rate	X	X	X	X	X		X
Body temperature	X	X	X	X	X		
Blood work-up (clinical routine)	X†	X†					X
Symptom questionnaire		X					
Echocardiography (standard clinical protocol)		X	(X)‡				(X)‡ day 30 (±48 hours)
NT-proBNP	X†	X	(X)‡				
Biobank (if separate consent)		X					
12-lead ECG	X†	X	(X)‡				
Chest X-ray	X†	X	(X)‡				
Exacerbation-specific treatment	X	X	X	X	X		
Complications and outcome	X	X	X	X	X	X	X
Spirometry, diffusion capacity, body plethysmography							X
6-minute walk test. COPD assessment test, mMRC							X

* After inclusion.

† When available.

‡ Only if positive in screening.

COPD, chronic obstructive pulmonary disease; GOLD, Global Initiative for Obstructive Lung Disease; mMRC, modified Medical Research Council Dyspnea Scale; NT-proBNP, N-terminal pro-B-type natriuretic peptide.

#### Baseline characteristics

Baseline characteristics include age, sex, body mass index, comorbidities, smoking status, baseline medication, presenting symptoms and signs, and are collected from interview with the patient and/or the patient’s medical chart. The time from admission (defined as arrival to hospital, either to emergency department or directly to hospital ward if bypassing emergency department) to inclusion is registered for all patients.

#### COPD characteristics

For all patients, COPD severity stage by GOLD criteria will be registered based on previous clinical records when available, and severity of exacerbation will be defined and registered according to the Rome criteria as suggested by GOLD.^17 22^ The patients’ COPD will be characterised at the time of inclusion and will then be further characterised in a stable state within 6 months after discharge. Specifically for classification of severity of AECOPD, the following variables are used: dyspnoea visual analogue scale from symptom questionnaire, respiratory rate, heart rate, oxygen saturation, CRP and arterial blood gas (table 3).^22^

**Table 3:** Severity of AECOPD

Mild (default)	Dyspnoea VAS <5. RR <24 breaths per minute. HR <95 beats per minute. Resting oxygen saturation ≥92% breathing ambient air (or patient’s usual oxygen prescription) AND change ≤3% (when known). CRP <10 mg/L (if obtained).
Moderate (meets at least three of five)	Dyspnoea VAS ≥5. RR ≥24 breaths per minute. HR ≥95 beats per minute. Resting oxygen saturation <92% breathing ambient air (or patient’s usual oxygen prescription) AND/OR change >3% (when known). CRP ≥10 mg/L. If obtained, arterial blood gas may show hypoxaemia (PaO_2_ ≤8.0 kPa) and/or hypercapnia (PaCO_2_ >6.0 kPa) but no acidosis (pH >7.35).
Severe	Arterial blood gas shows hypercapnia and acidosis (PaCO_2_ >6.0 kPa and pH <7.35).

Adapted from Celli *et al*^22^ (The Rome Proposal).

AECOPD, acute exacerbation of chronic obstructive pulmonary disease; CRP, C-reactive protein; HR, heart rate; RR, respiratory rate; VAS, visual analogue scale.

Also, the following COPD/exacerbation-specific characteristics (when available as part of clinical routine) are collected at the time of hospitalisation: chest CT, nasopharyngeal swabs for detection of viral infection, sputum sampling for culture, handheld spirometry for forced expiratory volume in 1 s, COPD assessment test, level of oxygen supply (baseline home oxygen and/or oxygen administered because of hypoxia during exacerbation) and treatment choices (inhalations, antibiotics, corticosteroids).

#### In-hospital follow-up

Blood pressure, heart rate, pulse oximetry, respiratory rate, body temperature and AECOPD-specific treatments (including detailed information on beta-2 agonist inhalation and other inhalation treatment) are registered daily until day 3 after inclusion. Complications (including date and approximate time) during hospitalisation are collected consecutively for all patients according to the following: acute respiratory failure/deterioration, AHF/cardiac decompensation, acute myocardial infarction, stroke, PE, peripheral embolisation, admission to intensive care unit, need for respiratory support, in-hospital cardiac arrest and in-hospital death.

#### Postdischarge follow-up

Echocardiography at day 30 (±48 hours) is performed after discharge or in hospital depending on length of stay (only screening-positive patients). In the stable state, the following characteristics from re-evaluation (as part of clinical routine after hospital care for AECOPD, according to hospital medical chart) are collected: postbronchodilator spirometry, diffusion capacity, static lung volumes (body plethysmography), 6-minute walk test, COPD assessment test, modified Medical Research Council Dyspnea Scale, oxygen saturation and/or blood gases and GOLD A, B, E classification. Also, all-cause and cardiovascular mortality within 1 year after inclusion will be registered (data from Swedish Cause of Death Register, National Board of Health and Welfare).

### Outcomes

#### Primary outcomes

1. Cumulative incidence of reversible RWMA or reversible left ventricular systolic dysfunction (as proxy for Takotsubo stunning) according to screening and follow-up echocardiography.
2. Clinical signs of AHF during hospitalisation (defined as Killip class >1, table 1, components of classification documented in hospital medical chart).

#### Secondary outcomes

1. In-hospital 3-point major adverse cardiac events (MACE): non-fatal myocardial infarction, non-fatal stroke or cardiovascular death (according to hospital medical chart).
2. Admission to intensive care unit (according to hospital medical chart).
3. In-hospital all-cause death (according to hospital medical chart).
4. Respiratory failure, acute or acute on chronic (hypercapnia with acidosis, PaCO_2_ >6.0 and pH <7.35, according to lab values in hospital medical chart).
5. ST elevation, ST depression, QT prolongation, T-wave inversion on ECG (ECG at admission and inclusion).
6. Level of NT-proBNP (obtained at admission and inclusion).
7. One-year all-cause and cardiovascular death (Swedish Cause of Death Register, National Board of Health and Welfare).

### Statistical plan and sample size

Inclusion is planned over approximately 3 years to estimate the cumulative incidence of Takotsubo stunning in patients hospitalised for AECOPD. The primary analysis will assess the association between a positive screening result and clinical manifestations of AHF using multivariable logistic regression. The model will adjust for relevant baseline confounders, with a significance threshold of α=0.05.

For the primary analysis, the required sample size is estimated at 150 patients, assuming a 1:10 ratio of positive to negative screening results. In previous research randomly investigating the correlation between clinical and echocardiographic signs of heart failure, clinical heart failure was present in approximately 50% of patients with echocardiographic left ventricular systolic dysfunction (LVEF <40%).^23^ Given the higher cut-off for systolic dysfunction in CATCH (LVEF <50%) and inclusion of newly detected RWMA as positive screening (ie, also in absence of reduced LVEF), together with the exclusion of patients with prior type 1 myocardial infarction or known persistent systolic dysfunction, we conservatively estimate the proportion of clinical heart failure in the positive screening group to approximately 30%. Accordingly, our sample provides 80% power to detect a difference in the incidence of clinical signs of AHF between the groups, assuming 30% in the positive screening group and 5% in the negative screening group with α=0.05.

Secondary and tertiary outcomes will be analysed using linear regression for continuous outcomes and logistic regression for dichotomous outcomes, in both unadjusted and adjusted models. A Cox proportional hazards model adjusted for baseline confounders will be applied to analyse 1-year mortality. These analyses are considered explanatory or hypothesis generating. Results will be reported as effect estimates with 95% CIs and unadjusted p values, with false discovery rate correction applied to account for multiple comparisons.^24^

Unadjusted and adjusted analyses (including covariates age and sex) will be presented as separate models. A sample size of 150 patients, with an assumed 1:10 ratio of positive to negative screening results, translates to a total number of 15 events. Given the relatively low number of expected events, all further adjustment will include only clinically meaningful covariates and will be presented as separate models.

### Study status

Inclusion started on September 2024 and to date (April 2026), 56 patients have been enrolled (of 150 patients intended for the primary analysis). Analysis is planned after end of inclusion.

### Scientific and clinical importance

The risk of both fatal and non-fatal cardiovascular disease is a major concern in patients with COPD, especially after a hospitalisation for AECOPD. This established excess cardiovascular risk after AECOPD is incompletely understood. A link seems to exist between AECOPD and Takotsubo stunning, which could be a contributing mechanistic explanation, supported by previous literature.^12 12^,^15 25^ Leading authors in the field of TS have specifically advocated further investigations of this association.^2^

This study is clinically relevant for two main reasons: (1) the cardiac condition may be masked by the respiratory symptoms and the clinical signs of AECOPD, leading to delayed or missed diagnosis of the cardiac component, (2) Takotsubo stunning with cardiac dysfunction may further exacerbate the patient’s condition, leading to prolonged time to recovery due to concomitant untreated AHF and an increased risk of severe cardiac complications.^2 5 15^

Another important aspect is the treatment for AECOPD, which may include treatment with high doses of beta-2 agonists. Catecholamines are part of the pathophysiological cause of Takotsubo stunning, and catecholaminergic drugs may further aggravate Takotsubo stunning. In fact, beta-2 agonists specifically have been shown to likely trigger Takotsubo stunning. Therefore, the routine treatment for AECOPD may be associated with a risk of harming patients with concomitant AECOPD and Takotsubo stunning.^5 6^

If successful, the CATCH study may improve the quality of care and outcomes for patients with AECOPD by highlighting the need to address AECOPD as a cardiopulmonary syndrome, rather than a purely pulmonary condition. In addition, the study may help guide optimisation of treatment strategies for AECOPD, for example, by providing insights into settings in which excessive use of beta-2 agonists may be inappropriate.

### Patient and public involvement

This study originates from the person-centred care context at Sahlgrenska University Hospital, where patient perspectives are a central part of everyday clinical work, in accordance with the hospital’s core value explicitly phrased in the hospital policy: ‘For the patient, with the patient’. Through ongoing dialogue with patients, previously unrecognised clinical needs and gaps in existing knowledge were identified, which influenced the study objectives. This is particularly relevant since the majority of investigators and collaborators in this study are also treating physicians for the included patients. Patients and the public were not otherwise involved in the study design or conduct.

Results of the study will be disseminated through scientific publications and presentations. In addition, findings will be communicated in formats accessible to patients and the public, including plain language summaries and dissemination through relevant clinical settings where patients receive care.

## Ethics and dissemination

A cooperation with Gothia Forum (regional unit for clinical research and trial support in Region Västra Götaland, Sweden) is established for monitoring/quality assurance. The CATCH study adheres to the principles of Good Clinical Practice and follows the Declaration of Helsinki. All participants provided verbal and written IC prior to inclusion. Study data is handled in accordance with applicable data protection regulations and stored on secure, access-controlled servers within Sahlgrenska University Hospital and Sahlgrenska Academy (University of Gothenburg, Sweden). The CATCH study is registered at ClinicalTrials.gov (NCT06597331), and findings from the study will be disseminated through peer-reviewed publications and conference presentations. No identifying information will be published.

## Data Availability

No data are available.
